# Predictors of methotrexate adherence and patient's awareness of it in rheumatoid arthritis and its effect on quality of life

**DOI:** 10.1080/20523211.2024.2365933

**Published:** 2024-07-18

**Authors:** Marwa Mohamed, Abd El-Maboud, Heba F. Salem, Mohamed N. Salem, Mahmoud Abo Elmaaty, Nermin Eissa, Amira S. A. Said, Raghda R. S. Hussein

**Affiliations:** a Clinical Pharmacist at Medical Administration, Fayoum University, Egypt; b Department of Clinical Pharmacy, Faculty of Pharmacy, Beni-Suef University, Cairo, Egypt; c Department of Pharmaceutics & Industrial Pharmacy, Faculty of Pharmacy, Beni-Suef University, Cairo, Egypt; d Department of Internal Medicine, Faculty of Medicine, Beni-Suef University, Cairo, Egypt; e Senior Clinical Pharmacist at the Children Cancer Hospital in Egypt, Beni-Suef University, Cairo, Egypt; f Department of Biomedical Sciences, College of Health Sciences, Abu Dhabi University, Abu Dhabi, United Arab Emirates; g Department of Clinical Pharmacy, College of Pharmacy, Al Ain University, Abu Dhabi, United Arab Emirates

**Keywords:** Adherence, knowledge, functional disability, methotrexate, rheumatoid arthritis

## Abstract

**Introduction:**

Adherence studies among rheumatoid arthritis (RA) patients, in Egypt and throughout the Middle East region, are lacking. This study aimed to evaluate methotrexate (MTX) adherence in Rheumatoid arthritis (RA) patients and to identify specific non-adherence predictors.

**Methods:**

A cross-sectional observational study included 300 RA patients who were administered MTX for at least one year. The survey was completed through direct interviews. The demographic patient data were collected (age, education, sex, work status, disease duration, duration of MTX administration and current dose). Patients' adherence to MTX predictors for non-adherence, MTX side effects and functional disability were assessed in the study.

**Results:**

Majority of respondents showed good MTX adherence, and more than 50% of patient’s experienced MTX side effects. A large percentage of participants showed low knowledge about MTX nature and side effects. Most participants reported no or some difficulty in quality of life-related activities and functional disability.

**Conclusion:**

MTX adherence and awareness were positively correlated to many variables, including, age, educational level and disease duration, which in turn has its positive impact on the patient's quality of life. Still, more research is needed to determine the impact of non-adherence on the patient's health outcomes.

## Background

Rheumatoid arthritis (RA) is an immunological disorder in which the membrane surrounding the joints gets inflamed (the synovium). Swelling, stiffness, as well as limited joint mobility, are common signs of RA. Moreover, RA can progress to functional impairment, and deformities, with a prevalence of 0.6% of the population (DiBenedetti et al., 2015). Women are 2–3 times more likely than men to get RA (DiBenedetti et al., 2015; Sakr et al., 2018). RA prevalence varies globally, with higher incidence rates in highly industrialised nations due to risk hazards associated with the environment, genetics, and demographics. Major hazards for RA encompass modifiable lifestyle factors and non-modifiable aspects like genetics and sex. It was reported that developed countries have the highest incidences of RA, followed by India and South American nations. Understanding the natural progression of RA can help predict how future changes in the global society and climate will impact the disease's prevalence (Finckh et al., 2022).

Pharmacological management of RA includes three medication classes: non-steroidal anti-inflammatory drugs (NSAIDs), corticosteroids, as well as disease-modifying antirheumatic drugs (DMARDs). While the first two classes of drugs have a quick onset of action, DMARDs often take weeks to months to show a clinical response (DiBenedetti et al., 2015). Methotrexate (MTX) is a typical first-line DMARD therapy for RA patients worldwide, however, MTX discontinuation and treatment changes still remain a considerable issue in managing RA patients. Gastrointestinal disturbances are one of the major reasons for MTX withdrawal, which may vary from nausea, vomiting, malaise to active gastric ulcers. Symptoms can be managed through dosage reduction, 12-hour dose distribution, weekly dose division, evening/parenteral utilisation, or symptomatic therapy with metoclopramide (Albrecht & M ler-Ladner, 2010).

In RA patients, early initiation of MTX can assist in managing joint destruction as well as reduce disease progression (Malik & Ranganathan, 2013). However, the prevalence and severity of MTX adverse effects are proportional to its dose and frequency of administration (Schnabel et al., 1994).

Although MTX's long-term effectiveness, tolerance and safety have all been extensively demonstrated, patient adherence may be influenced by MTX clinical response as well as the frequency of side events (Kremer & Phelps, 1992; Malik & Ranganathan, 2013; Rau et al., 1997; Schnabel et al., 1994,1996). In addition, RA disease, duration, aggressiveness and comorbidities can all impact patient adherence (Schnabel et al., 1996).

Despite various systematic and comprehensive studies having been conducted to investigate adherence to DMARDs, there is a deficiency of data explaining non-adherence to MTX administration in RA patients (Curtis et al., 2016; Harrold & Andrade, 2009; Hope et al., 2016; Rauscher et al., 2015; Salt & Frazier, 2010). Adherence is commonly characterised as individual patient behaviour, where it is considered a balance between patients' beliefs about the need for the therapy and their fears about its adverse effects.

It has been stated that, in comparison to other chronic diseases such (asthma and diabetes), adherence to RA treatment is not clear (Gadallah et al., 2015; Pascual-Ramos et al., 2009). Medication adherence is commonly defined as ‘the extent to which patients receive medications as directed by their physicians’, however, there is no ideal standard for assessing drug adherence in RA patients (Ko et al., 2020).

Poor adherence to MTX as a typical first-line DMARDs therapy has been linked to several criteria, including confidence in the medicine, perceptions of the illness, self-efficacy, demographic, and adherence-related psychological and clinical factors (Brus et al., 1999; Suh et al., 2018). Rheumatoid arthritis is usually not aware of MTX side effects, particularly the most threatening ones (haematologic and hypersensitivity pneumonitis). Lack of MTX awareness is even more pronounced in elderly patients and those with low education levels (Fayet et al., 2016).

Several different methods have been used to assess MTX adherence including self-reports, pill counts, pharmacy records, plasma drug levels and electronic monitoring. Each of these methods has advantages and disadvantages (de Klerk et al., 2003a, 2003b). Recent publication demonstrated a new pharmacokinetic model that potentially used urinary MTX (MTXu) as a new objective test for MTX adherence. Although urinary MTX could effectively differentiate truly unresponsive patients to MTX from those who are unresponsive because of bad observance, this method only assesses adherence with the previous dose. while randomly missed doses will not be identified in that manner (Geoffroy et al., 2023). Furthermore, self – reporting surveys may be clinically relevant for the quick identification of adherence (Ceranic et al., 2023). Moreover, the previous literature review has shown that steroid administration may improve adherence, where adherence was significantly higher in a group of patients receiving steroids since the absence of medication can result in a considerable exacerbation of symptoms in the most active stage of the disease (Van Den Bemt et al., 2012) . In addition, Patient education programmes, non-profit organisations, and government policies that promote affordable and readily available healthcare can help improve the quality of life as well as medication adherence in RA patients (Ahmed et al., 2023). Arthritis Research UK (previously the Arthritis Research Campaign) has created patient information leaflets on MTX to promote awareness, education, and safety (Sowden et al., 2012). Furthermore, the NPSA, National Institute for Clinical Excellence, and Evidence provide crucial recommendations for avoiding MTX-related damage (Innes et al., 2022; Visser et al., 2009). Also, the weekly text messages showed evidence of a positive impact in patients taking MTX for RA on adherence to MTX (Mary et al., 2019). In societies with high educational levels, introducing ChatGPT-like technologies to bridge the communication barriers between RA patients and healthcare providers may be very promising (Chen et al., 2024).

The aim of this research was to investigate patients’ self-reported adherence to MTX and to assess causes for non-adherence relying on patient-experienced side effects, awareness of the drug usage, satisfaction, and activities of daily living (ADL) health-related quality of life.

To our data, there is a deficiency in studies assessing the RA patients’ adherence to MTX and its effect on their quality of life,

## Methods

### Study design

An observational cross-sectional clinical study was conducted on Egyptian RA patients, in outpatient clinics in the rheumatology department of the Faculty of Medicine Beni-Suef University Hospitals. This study was conducted over a period of one year, via direct interviews from February 2021 to February 2022.

All methods were carried out in accordance with relevant guidelines and regulations.

The study was approved by the research ethics committee of the Faculty of Medicine, Beni-Suef University (FM-BSU REC), in compliance with the guidelines of the Declaration of Helsinki. Approval was registered with the registration number FMBSUREC/05032019. Written informed consent was obtained from all patients prior to their enrollment.

In addition, the study was registered in Clinical trials. gov with number (NCT 05535686).

## Study population

### Inclusion criteria

The survey participants were adult patients (≥18 years) diagnosed with RA in accordance with 2010 ACR/EULAR classification criteria and were treated with either MTX alone or in combination with other DMARDs from (1-5) years. All patients signed a written informed consent form prior to inclusion. The clinical consultations are provided for free. There are no fees for consulting a physician. Previously, university hospitals provided free prescription drugs, however, this is no longer the case. The sample size was calculated using Open-Epi, Version 3, open-source calculator based on the assumption that 90% of patients would have good adherence (Gadallah et al., 2015); at least 139 patients should be enrolled to the study to achieve a 95% confidence level and 5% margin of error. A 300 RA patients participated in this study.

### Exclusion criteria

Patients who were on MTX for >5 years, or had complications (liver or kidney disease, diabetes, hypertension, hereditary alopecia, leukopoenia, significant anaemia, peptic ulcer or any GIT problems,) were excluded from the current study.

### Questionnaire design

A self-administered questionnaire was designed and adapted based on a literature review (de Klerk et al., 1999, 2003a, 2003b).

The questionnaire was distributed by researchers who approached and interviewed each participant to explain the study objectives and obtain their consent. The structured self-administered questionnaire aimed to assess patients' adherence, awareness, side effects reported and quality of life with MTX utilisation.

The questionnaire was validated and examined for content relevance. Face validity was assessed using convenience sampling of the previously validated Compliance Questionnaire Rheumatology (CQR), which assessed patient adherence towards MTX. CQR is a 19-item survey constructed from patient interview sessions and interviewing focus groups that summarise statements made by individual patients concerning their drug-taking behaviour (de Klerk et al., 1999, 2003a, 2003b). Participants were asked to evaluate the clarity of the questions and their relevance to the study objectives. The final structure of the questionnaire was amended as required.

The questionnaire had 5 sections; for more details (see Supplemental Material).

The health assessment questionnaire (HAQ) is a reliable way of assessing disability covering 20 distinct functions organised into categories: dressing and grooming, arising, eating, walking, personal hygiene, reaching, gripping and other activities. The response scores towards functional disability range from (without any difficulty) to (unable to do) on a 5-point Likert scale (Bruce & Fries, 2003).

Regarding the adherence towards methotrexate and awareness, a five-point Likert scale (strongly agree, agree, undecided, disagree, strongly disagree) was used. Responses towards quality of life were represented by a five-point Likert scale ‘without any difficulty, with some difficulty, undecided, with much difficulty, unable to do’ A score of ‘1’ point was assigned to ‘strongly disagree’ or ‘without any difficulty,’ while scores of ‘5’ were assigned to responses of ‘strongly agree.’ Or unable to do. Negatively worded statements (that did not reflect a positive attitude) were reverse-coded. The total score for each section was calculated by the simple addition of the responses from the study population.

### Statistical analysis

We calculated a total score for each construct included in the questionnaire. Statistical analysis was performed using IBM SPSS version 26.0 (IBM Corp. Released 2019. IBM SPSS Statistics for Windows, version 26.0 Armonk, NY: IBM Corp). Descriptive values were presented as average values with standard deviation or as median with an interquartile range (25–75th percentile). Distribution was verified by the Kolmogorov–Smirnov test. Attribute variables were presented in the form of frequencies of individual categories. Statistical significance was estimated by the Chi-square test or Fisher's exact test for attributive variables. Continuous variables were tested using Student's t-test for (in) dependent samples or nonparametric alternative Mann–Whitney test and Wilcoxon test. Predictive value exploration for the chosen variables in the assessment of adherence to MTX, awareness and quality of life-related activities in the group of patients with RA was conveyed by univariate and multivariate linear regression analyses. A *p*-value less than 0.05 for variables in the univariate analysis was used for inclusion in the multivariate analysis. The results of the linear regression analysis were presented as Beta (B coefficient of X) with a 95% confidence interval (B (95% CI)). A *p*-value less than 0.05 was considered statistically significant for all analyses.

The internal consistency reliability of the three used questionnaires (extent to which individual items are consistent with each other in the same dimension and reflect a single underlying concept) was assessed by the calculation of Cronbach's alpha for each dimension and overall. A minimum alpha value of 0.70 is generally considered acceptable. Overall Cronbach's alpha for Patients' adherence towards MTX was (0.715), for Patients' awareness towards MTX was (0.827) and for quality of life-related activities was (0.926). The Statistical Package for the Social Sciences (SPSS), version 20.0, for Windows was used. The tests were two-tailed, and *p*-values < 0.05 were considered to indicate statistical significance.

## Results

### Descriptive and univariate analysis

This study included 300 RA patients and the participants’ demographic details were summarised in (Table 1). As shown in Table 1, the study participants were 89% female. The mean age of participants was 46 years, and up to 39.3% were above 50 years old. In addition up to 64% of participants were unemployed and about 38.3% were highly educated. Up to 55% of participants used more than 12.5 mg per week of methotrexate, with mean Disease duration (4 ± 1) years and mean Treatment duration (2 ± 1) years.

**Table 1.** Demographic characteristics of recruited participants (*N* = 300).

Variable		Measurement	
Age (years) (*n*, %)	≤ 50 years old	182	60.7%
	>50 years old	118	39.3%
Age in yrs (mean ± SD) 46.1 ± 12.3			
Gender (*n*, %)	Female	267	89.0%
	Male	33	11.0%
Education (*n*, %)	Low	94	31.3%
	Intermediate	91	30.3%
	High	115	38.3%
Work status (*n*, %)	Without work	192	64.0%
	Clerks	66	22.0%
	Manual labour worker	29	9.7%
	Retired	13	4.3%
Methotrexate dose (*n*, %)	Less than 12.5 mg per week	26	8.7%
	12.5 mg per week	109	36.3%
	More than 12.5 mg per week	165	55.0%
	Monthly dose	0	0.0%
Disease duration (years) (mean ± SD)		4 ± 1	
Treatment duration (years) (mean ± SD)		2 ± 1	

Demographic and clinical factors were examined for associations with medication adherence, awareness, and impaired quality of life as shown in Table 2.

**Table 2.** Association between patients' characteristics and domain scores (*n* = 300).

Variable		Adherence Mean ± SD	*p*-value	Awareness Mean ± SD	*p*-value	Impaired Quality of Life Mean ± SD	*p*-value
Age	≤50 yrs.	18.75 ± 3.28	0.43	17.92 ± 5.62	0.52	18.98 ± 9.26	0.001
	> 50 yrs.	18.42 ± 3.98		17.50 ± 5.60		24.91 ± 9.44	
Gender	Male	18.64 ± 3.60	0.85	17.65 ± 5.60	0.071	21.67 ± 9.73	0.34
	Female	18.52 ± 3.36		18.64 ± 5.66		18.42 ± 9.61	
Education	Low	18.11 ± 3.88	0.201	18.05 ± 5.70	0.053	23.27 ± 9.63	0.031
	Intermediate	19.02 ± 3.52		18.88 ± 4.98		21.33 ± 9.47	
	High	18.73 ± 3.30		16.63 ± 5.83		19.70 ± 9.87	
Work status	without work	18.60 ± 3.70	0.178	17.93 ± 5.64	0.174	21.66 ± 9.78	0.501
	Clerks	18.95 ± 3.24		17.42 ± 5.86		21.08 ± 10.20	
	Manual labor worker	18.93 ± 3.36		18.72 ± 4.93		19.07 ± 8.30	
	Retired	16.62 ± 3.23		14.77 ± 4.51		22.38 ± 10.42	
Disease duration	≤3 yrs.	18.78 ± 3.68	0.559	18.25 ± 6.11	0.251	18.71 ± 9.16	0.001
	>3 yrs.	18.53 ± 3.51		17.47 ± 5.29		22.82 ± 9.80	
Methotrexate dose	<12.5 mg per week	18.69 ± 3.38	0.973	20.38 ± 5.27	0.017	18.00 ± 8.95	0.137
	12.5 mg per week			18.06 ± 5.64		21.03 ± 9.65	
	> 12.5 mg per week	18.66 ± 3.66		17.14 ± 5.53		22.02 ± 9.88	

This study has reported females to have more mean ± SD awareness (18.64 ± 5.66 vs 17.65 ± 5.60) and less mean ± SD impaired QOL (18.42 ± 9.61 vs21.67 ± 9.73) compared to males. However, all results failed to reach a significant difference.

Our study also reported that patients with intermediate educational levels showed the highest mean score of MTX adherence, and awareness and the least impaired QOL towards MTX. On the other hand, patients with low educational levels showed the highest mean ± SD score of impaired QOL of 23.27 ± 9.63vs 21.33 ± 9.47 and 19.70 ± 9.87 for the high and intermediate educational levels, respectively.

Patients taking >12.5 mg/wk of MTX showed the highest mean score of impaired QOL (22.02 ± 9.88) compared to those receiving 12.5 mg/wk (21.03 ± 9.65) or <12.5 mg/wk (18.00 ± 8.95)

**Figure 1.**
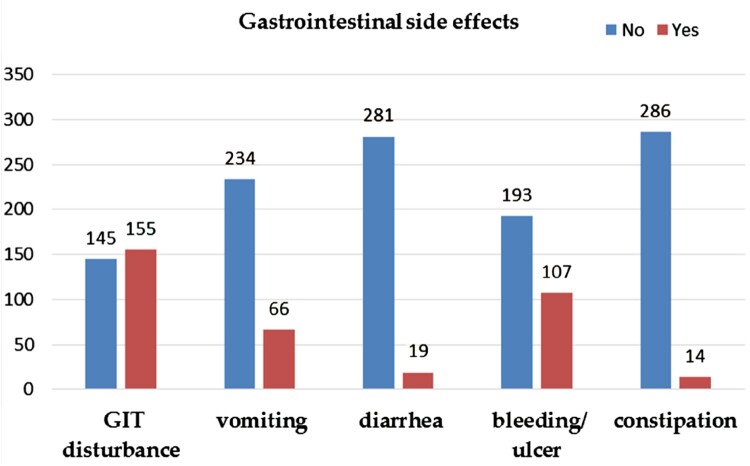
Reported gastrointestinal side effects (GIT disturbance, nausea, vomiting, bleeding/ulcer and constipation) by methotrexate users.

As shown in Figure 1, nausea was the most common side effect experienced by patients with utilisation of MTX. However, other side effects were also reported including; vomiting, diarrhoea, bleeding, or constipation.

The association between reported side effects and methotrexate adherence and awareness and impaired QOL were investigated and illustrated in Table 3.

**Table 3.** Association between reported side effects and methotrexate use (*n *= 300).

Question		Adherence	*p*-value	Lack of Awareness	*p*-value	Impaired Quality of Life (QOL)	*p*-value
		Mean ± SD		Mean ± SD		Mean ± SD	
Nausea	No	18.31 ± 3.60	0.142	16.75 ± 6.10	0.003*	20.20 ± 9.58	0.056
	Yes	18.92 ± 3.52		18.70 ± 4.94		22.35 ± 9.83	
Vomiting	No	18.58 ± 3.60	0.672	17.09 ± 5.84	0.000*	20.29 ± 9.40	0.001*
	Yes	18.79 ± 3.45		20.11 ± 3.89		24.92 ± 10.21	
Diarrhoea	No	18.62 ± 3.59	0.939	17.85 ± 5.64	0.284	21.24 ± 9.75	0.610
	Yes	18.68 ± 3.35		16.42 ± 5.00		22.42 ± 9.97	
Bleeding/ulcer	No	18.39 ± 3.62	0.135	17.26 ± 5.84	0.041*	20.31 ± 9.56	0.017*
	Yes	19.04 ± 3.45		18.64 ± 5.07		23.12 ± 9.89	
Constipation	No	18.58 ± 3.59	0.388	17.73 ± 5.64	0.755	21.38 ± 9.81	0.568
	Yes	19.43 ± 3.13		18.21 ± 5.15		19.86 ± 8.83	
Do you plan to stop the treatment if you get pregnant?	No	18.51 ± 3.59 3.59	0.219	17.82 ± 5.57 5.57	0.684	21.88 ± 9.62 9.62	0.030*
	Yes	19.17 ± 3.42 3.42		17.47 ± 5.82 5.82		18.68 ± 10.04 10.04	
Elevated liver enzymes	No	18.57 ± 3.60 3.60	0.419	17.72 ± 5.67 5.67	0.756	21.07 ± 9.78 9.78	0.172
	Yes	19.14 ± 3.21 3.21		18.07 ± 4.99 4.99		23.71 ± 9.35 9.35	
Impaired kidney functions	No	18.63 ± 3.59 3.59	0.955	17.59 ± 5.68 5.68	0.049*	20.99 ± 9.63 9.63	0.027*
	Yes	18.58 ± 3.27 3.27		20.21 ± 3.74 3.74		26.11 ± 10.53 10.53	
Headache	No	18.60 ± 3.61 3.61	0.652	17.61 ± 5.68 5.68	0.042*	20.93 ± 9.74 9.74	0.014*
	Yes	18.95 ± 3.05 3.05		19.64 ± 4.16 4.16		26.14 ± 8.84 8.84	

Note: *mean significance at *p* less than 0.05.

Significant lower Impaired QOL (*p* <0.05) was reported in those patients who reported nausea, vomiting, bleeding ulcer, impaired kidney functions and headache. However, adherence was not significantly affected by other variables as indicated in Table 3.

Patients’ adherence towards MTX use is summarised in Table 4.

**Table 4.** Patients' adherence towards methotrexate use (n = 300).

Questions	Strongly Disagree No (%)	Disagree No (%)	Undecided No (%)	Agree No (%)	Strongly Agree No (%)
You use methotrexate regularly as prescribed by a physician	0 (0%)	28(9.3%)	3 (1%)	82 (27.3%)	187 (62.3%)
You dare missing a methotrexate dose	150 (50%)	52 (17.3%)	4 (1.3%)	67 (22.3%)	27 (9%)
You use folic acid daily	59 (19.7%)	70 (23.3%)	107 (35.7%)	64 21.3%	0 (0%)
If you don't take the dose regularly, then the inflammation returns after a long duration	114 (38.0%)	45 (15%)	21 (7%)	47 (15.7%)	73 (24.3%)
Symptoms recurrence after stopping treatment	65 (21.7%)	29 (9.7%)	36 (12%)	35 (11.7%)	135 (45%)
Available in the pharmacy	35 (11.7%)	71 (23.7%)	38 (12.7%)	123 (41%)	33 (11%)
Total score	Mean = 18.62 ± 3.56 (11-29)				

The Results showed that most patients used MTX regularly. Many patients (45%) agreed that symptoms usually recur after stopping MTX treatment. Most of them reported that recurrence of inflammation is not after a long duration if they missed administrating the dose regularly. Finally, most participants agreed about drug availability in general pharmacies. Patients' awareness towards MTX use is summarised in Table 5.

**Table 5.** Patients' awareness towards methotrexate use (*n* = 300).

Question	Strongly Disagree	Disagree	Undecided No	Agree	Strongly Agree
	(%)	(%)	(%)	(%)	(%)
Methotrexate has a delayed onset of action, the dose has a long time to get its effect	180 (60%)	32 (10.70%)	25 (8.3%)	21(7%)	42 (14%)
You may stop treatment when symptoms disappear or adjust the dose according to the severity of the pain	87 (29%)	63 (21%)	14 (4.7%)	75 (25%)	61 (20.3%)
You may not take the dose if you feel you can miss it	52 (17.3)%	42 (14%)	3 (1%)	90 (30%)	113 (37.7%)
You do not expect miracles from your treatment	72 (24%)	105 (35%)	31(10.30%)	58 (19.3%)	34 (11.30%)
After every methotrexate dose, you experience an increase in hair fall and hair loss with long-term use	107 (35.6%)	45 (15%)	11 (3.7%)	74 (24.7%)	63 (21%)
You prefer to take another drug to treat R.A other than methotrexate even if it may be less effective than methotrexate	49 (16.3)%	52 (17.3%)	13 (4.3%)	74 (24.7%)	112 (37.3%)
Total score	Mean 17.75 ± 5.60 (6-30)				

Majority of patients disagree that there was a delayed onset of action of MTX use. In addition, a significant number of participants disagree that treatment should be stopped on the disappearance of symptoms or adjust the dose according to the severity of the pain.

In addition, a large percent of patients strongly agreed to miss the dose if they felt they could miss it.

Moreover, the majority of them disagree that they knew that one of the side effects of MTX may cause an increase in hair fall and hair loss with long-term use.

Many participants (37.3%) strongly agreed to prefer taking another drug to treat RA other than MTX even if it may be less effective than MTX.

The patients reported quality of life-related activities is summarised in Table 6.

**Table 6.** Reported quality of life-related activities (*n* = 300).

Question	Without Any Difficulty	With Some Difficulty	Undecided No	With Much Difficulty	Unable to Do
	(%)	(%)	(%)	(%)	(%)
Dress yourself, including tying shoelaces and doing buttons	141 (47%)	116 (38.7%)	0 (0%)	41 (13.7%)	2 (0.7%)
Shampoo your hair	143 (47.7%)	117 (39.0%)	0 (0%)	37 ()12.3%	3 (1%)
Arising to get in and out of bed	111 (37.0%)	118 (39.3%)	1(0.3%)	67 (22.3%)	3 (1%)
Eating (cutting your meat /Lift a full cup or glass to your mouth/Open a new milk carton)	67 (22.3%)	96 (32.0%)	2 (0.7%)	90 (30.0%)	45 (15%)
Arising stand up straight from an armless straight chair	123 (41.0%)	70 (23.3%)	1(0.3%)	60 (20.0%)	46 (15.3%)
Walk outdoors on flat ground	75 (25.0%)	100 (33.3%)	0 (0%)	101 (33.7%)	24 (8%)
Bend down to pick up clothing from the floor	83 (27.7%)	104 (34.7%)	0 (0%)	78 (26.0%)	35 (11.7%)
reach object from above the head	112 (37.3%)	104 (34.7%)	1(0.3%)	77 (25.7%)	6 (2%)
climbing stairs	54 (18.0%)	111 (37.0%)	1(0.3%)	99 (33.0%)	35 (11.7%)
Total score	Mean = 21.31 ± 9.75(9–45)				

Majority of participants reported no or some difficulty in quality of life-related activities except walking outdoors.

### Multivariate analysis

From our logistic regression analysis, as presented in Table 7, we confirmed that predictors towards adherence to MTX were higher education, good awareness, and better quality of life.

**Table 7.** Predictors of adherence towards methotrexate (*n* = 300).

Model	Independent Variable	*B*	*t*	*p*-value	95.0% Confidence Interval for B	
					Lower Bound	Upper Bound
1	(Constant)	12.181	7.557	0.000	9.008	15.353
	Age	0.013	0.732	0.465	−0.022	0.049
	Gender	−0.510	−0.833	0.406	−1.716	0.695
	Education (high)	0.590	2.197	0.029*	0.061	1.118
	work status	−0.401	−1.478	0.141	−0.934	0.133
	Disease duration	0.150	1.041	0.299	−0.134	0.434
	Treatment duration	0.006	0.040	0.969	−0.299	0.311
	Methotrexate dose	0.517	1.727	0.085	−0.072	1.106
	Lack of awareness	0.312	8.784	<0.001*	0.242	0.382
	Impaired quality of life	−0.075	−3.466	0.001*	−0.117	−0.032

Note: *denotes *p* less than 0.05.

Table 8 shows that predictors of lack of awareness were associated with lower level of education, lower treatment duration, lower MTX use, and impaired quality of life.

**Table 8.** Predictors of lack of awareness towards methotrexate (*n* = 300).

Model	Independent Variable	B	t	*p*-value	95.0% Confidence Interval for B	
					Lower Bound	Upper Bound
1	(Constant)	8.498	3.320	0.001*	3.459	13.536
	Age	−0.032	−1.225	0.222	−0.085	0.020
	Gender	1.553	1.724	0.086	−0.220	3.327
	Education (high)	−0.915	−2.308	0.022*	−1.695	−0.135-
	work status	0.382	0.951	0.342	−0.408	1.172
	Disease duration	−0.079	−0.371	0.711	−0.500	0.341
	Treatment duration	−0.844	−3.780	<0.001*	−1.284	−0.405
	Methotrexate dose	−1.411	−3.233	0.001*	−2.270	−0.552
	Adherence	0.682	8.784	<0.001*	0.529	0.835
	Impaired quality of life	0.151	4.844	<0.001*	0.090	0.212

Note: *indicates variables with significant *p*-value less than 0.05.

In addition, as shown in Table 9, predictors of impaired quality of life were age, those without work, increased disease duration, increased MTX dose, lack of adherence and lack of awareness.

**Table 9.** Predictors of impaired QOL towards methotrexate (*n* = 300).

Model		*B*	*t*	*p*-value	95.0% Confidence Interval for B	
					Lower Bound	Upper Bound
**1**	(**Constant**)	**4**.**875**	**1**.**026**	0.306	−4.480	14.231
	Age	0.231	4.972	<0.001*	0.140	0.323
	Gender	−2.981	−1.814	0.071	−6.217	0.254
	Education	0.062	0.085	0.932	−1.375	1.499
	work status	−1.514	−2.078	0.039*	−2.947	−0.080
	Disease duration	1.025	2.660	0.008*	0.266	1.783
	Treatment duration	0.691	1.661	0.098	−0.128	1.509
	Methotrexate dose	2.885	3.639	<0.001*	1.324	4.445
	Adherence	−0.542	−3.466	0.001*	−0.850	−0.234
	Lack of awareness	0.503	4.844	<0.001*	0.299	0.708

Note: Bold value that affect quality of life and have significant *p*-value. *with significant *p*-value less than 0.05.

## Discussion

To our knowledge, this is the first investigation thoroughly examining the correlation between adherence rate, patient awareness and impaired quality of life in Egyptian RA patients utilising MTX as a monotherapy or in combination therapy using a structured designed questionnaire. Considering the hypothesis of earlier studies, demographic and clinical factors have been reported to be risk factors for medication nonadherence. The predictive potential of demographic and clinical parameters were investigated for relevant correlations with medication nonadherence, awareness towards MTX and functional disability which were related to quality of life in RA patients.

### Demographic variables and clinical factors

Several demographic variables and clinical factors were associated with medication adherence, awareness, and impaired quality of life. These included (age, gender, education, work status, MTX dose, disease duration and treatment duration). Our results reported that patients <50 years old had a high adherence rate, awareness and preferable quality of life towards MTX in comparison with elderly patients (>50 years). This finding agrees with a previous study which reported younger patients to show more adherence with RA treatment (Gadallah et al., 2015). However, other studies have reported otherwise that older age is related to better drug adherence (Park et al., 1999; Tuncay et al., 2007; Viller et al., 1999). Similarly, several studies also confirmed that older age was associated with a higher adherence rate to DMARDs (Müller et al., 2012; Park et al., 1999; Ragab et al., 2017; Tuncay et al., 2007). It has been observed that younger patients who lead a busy schedule are more inclined to disregard doctor's orders (Park et al., 1999; Tuncay et al., 2007).

In the present study, female patients were more adherent to MTX than male patients, however, in the previous research, sexual identity was not really related to treatment adherence in RA patients (Müller et al., 2012; Pasma et al., 2013).

Our study confirmed that educational level had a significant effect on patient adherence and awareness towards MTX. This study reported that patients with intermediate educational level had the highest score of adherence and awareness towards MTX. On the other hand, patients with low educational levels had the highest score of impaired quality of life. This finding is consistent with a prior study that reported that treatment non-adherence was related to illiteracy (Sharma et al., 2015). However, other studies found no association between the level of education and treatment adherence (Müller et al., 2012; Pasma et al., 2013; Ragab et al., 2017).

The current study reported that the disease duration was inversely proportional to the patient's adherence. It has been noted that patients with a disease duration of less than 3 years had good adherence and awareness towards MTX and less impaired QOL in comparison to patients with a disease duration of more than 3 years. This is in agreement with previous studies that reported that increased disease duration was significantly related to low adherence (Gadallah et al., 2015). Similarly, other studies' results have consistently shown that adherence to medications decreases with time (de Klerk et al., 2003a, 2003b; van den Hoogen et al., 2009), however, on the other hand, other studies reported no association between the disease duration and adherence to anti-rheumatic drugs (Müller et al., 2012; Ragab et al., 2017; Tuncay et al., 2007).

Our results have reported that patients taking higher MTX doses (>12.5 mg per week) showed the highest impaired quality of life and lowest awareness and adherence towards MTX. This could be attributed to the fact that patients who are prescribed higher doses may be probably out of remission and having an active RA disease. In contrast, another study found that a higher dose was associated with better persistence (Aletaha & Smolen, 2002). However, the most studies could not find an independent association between MTX dose and adherence (de Ideguchi et al., 2007; Salaffi et al., 1997; de Thurah, Nørgaard, Harder, & Stengaard-Pedersen, 2010). As a matter of fact, several research articles have revealed that medication adherence may not be significantly impacted by demographic factors (Tuncay et al., 2007; Van Den Bemt et al., 2012; van den Hoogen et al., 2009; Wong & Mulherin, 2007).

### Medication adherence measures

Currently, there is no gold standard approach for evaluating patients' drug adherence, therefore, the great variability of results may be due to the differences in methods used to assess adherence (Sutton et al., 2014). Most publications on medication adherence which included RA patients had described adherence as taking 80% or more of the recommended drug throughout the study duration (de Klerk et al., 2003a, 2003b; Dunbar-Jacob et al., 2004). Adherence can also be considered as a balance between patients' concerns about the treatment's negative effects and their ideas regarding its necessity (de Thurah et al., 2010). One study revealed that adherence was significantly influenced by the patient's perceptions about their medications and reported that moderately adherent responders had higher beliefs in the necessity and advantages of RA medications than did low-adherent patients (Rob Horne et al., 2005).

Another study indicated that patients who strongly believed that taking medication is crucial for maintaining their health were more likely to be more adherent to medications (Robert Horne et al., 1999). However, these studies employed a multitude of medication adherence measures and diverse research methodologies and were unable to detect recurrent risk factors for non-adherence to medication.

In this study, majority of patients used MTX regularly as prescribed and did not dare missing the dose due to fearing symptoms recurrence after stopping treatment. Another advantage which is contributing to MTX's good adherence is the availability of MTX in the governmental pharmacies. The previous study from Brazil has related patient non-adherence to the lack of MTX availability at the local pharmacy (Brus et al., 1999; Simpson, 2006).

In addition, many patients in the present study disagreed about using folic acid daily, which did not seem to affect our participants' adherence level towards MTX. However, it has been demonstrated that taking folic acid significantly reduced the overall withdrawals from MTX (Shea et al., 2014). Moreover, the general lack of documentation of folate supplementation in the reviewed studies, commenting on the potential impact of this variable on adherence is challenging. Even so, a previous study confirmed a positive correlation between folic supplementation and adherence (Hoekstra et al., 2003). Despite this, other studies reported no correlation (Bernatsky & Feldman, 2008; Ideguchi et al., 2007).

Our results have reported poor knowledge levels among older patients and those with a low educational level. This agrees with a previous study that also reported that patient awareness towards the MTX is associated with age and educational level (Fayet et al., 2016). Therefore, it is important to regularly assess patient knowledge towards MTX and provide support using the various therapeutic education technologies available, particularly in cases where patients are elderly or have limited education.

Functional disabilities were significantly associated with patients’ quality of life, contributing to high unemployment, and decreased productivity. In general, functional disability tended to increase with high disease activity and joint mobility limitations. Previous studies have revealed that RA patients with a low quality of life had a higher risk of functional disability (Gong & Mao, 2016; Ji et al., 2017; Wallman et al., 2016). Furthermore, several findings have indicated that during the first 18 months of RA onset, patients face a significant burden on functional ability (Kosinski et al., 2002).

RA is a chronic and lifelong medical condition, therefore therapeutic attention has shifted away from symptomatic relief to improving or trying to restore quality of life as a critical therapeutic goal (Fries et al., 1980; Haroon et al., 2007).

This study demonstrated that most participants reported no or some difficulty in quality of life-related activities and functional disability except walking outdoors.

To specify which dependent variables were most clearly correlated to functional disability, logistic regression analysis was done.

Our study revealed that there was a significant association between medication adherence and functional disability, which might explain that RA patients with reduced medication adherence, could have high disease activity. This also explained why patients taking high doses of MTX had with highest impaired quality of life in comparison with others which taking low doses of MTX. This comes in accordance with other studies (Häkkinen et al., 2005; Imran et al., 1969; Ji et al., 2017; Karpouzas et al., 2012; Overman et al., 2012; Pascual-Ramos et al., 2009; Pincus et al., 1989; Ruban et al., 2016; Simón-Campos & Padilla-Hernández, 2008; Sokka et al., 2000; Welsing et al., 2001).

Additionally, this study, reported that impaired quality of life was age-related. This comes in line with previous studies which reported the same results (Ruban et al., 2016; Simón-Campos & Padilla-Hernández, 2008). Furthermore, our study reported that increased disease duration was also significantly associated with impaired quality of life-related functional disability in patients with RA, which was in line with previous studies (Ji et al., 2017; Ruban et al., 2016; Zhao et al., 2015).

This study has its limitations because of the heterogeneity of the existing data, as most of our respondents were females and in younger ages, it was challenging to generalise the findings to the typical RA patient population.

Another limitation is the study bias selection, since all included participants were recruited from the same hospital and had an opportunity for more frequent follow-ups, which could have diminished the inclusion of non-adherent patients. Furthermore, utilising self-assessment instruments may result in overestimation of adherence as a result of social desirability and giving adjusted answers. This possibility leaves self-report questionnaire scores as ‘subjective,’ in contrast to observer-derived ‘objective’ data, such as laboratory tests and radiographs, which involve high-technology sources and health professional observers, without any input from the patient.

The research study was conducted in a non-profit government hospital that provides free treatment to all patients under the health insurance system, where medication costs were not considered a barrier to adherence. As a result, the findings might not be relevant to more representative populations of patients, wherever cost considerations, cultural differences, as well as more extensive or more varied disease burdens have been prevalent.

Relevant to clinical practice, the findings of this study ought to alert healthcare providers to the necessity for more robust patient education about MTX's potential severe adverse effects and to serve as a basis for designing a therapeutic education programme. Furthermore, this study aimed to investigate the prevalence of functional disability in Egyptian RA patients. Functional impairment is an important outcome indicator among RA patients since it can lead to unemployment, reduced productivity, poverty, and a lower quality of life.

Finally, This questionnaire, was created to fulfil an immediate local need, however, it can be improved further through conducting validation tests on bigger and more diverse groups of people. Therefore, this questionnaire can be considered as the initial phase towards constructing patient-reported outcomes.

## Conclusion

This study showed that treatment adherence and awareness were positively correlated to many variables, including, age, educational level and disease duration, which in turn has its positive impact on the patients' quality of life. Adherence to MTX is extremely variable in RA patients. To appraise evidence-based intervention strategies, more research is needed to determine the impact of non-adherence on health outcomes and to distinguish independent predictors of non-adherence. Although adherence evaluation is not standardised, however, trying to assess adherence, and causes of non-adherence are crucial from a clinical standpoint and should be part of standard clinical practice.

Realising the crucial role of pharmacists in assessing adherence and simplifying drug regimens, skilled and motivated pharmacists may be particularly promising to promote MTX medication adherence and persistence. Pharmacists medications' knowledge and proximity to patients should provide patients with proper medication counselling, and provide the needed lifestyle modifications and support that are needed by patients for a more improved quality of life.

## Supplementary Material

Supplemental Material
