# Supplementary material for: Predictors of methotrexate adherence and patient's awareness of it in rheumatoid arthritis and its effect on quality of life
Source: J Pharm Policy Pract. 2024 Jul 18;17(1):2365933. doi: 10.1080/20523211.2024.2365933 (PMC11259066; doi:10.1080/20523211.2024.2365933)
Supplement: Supplemental Material [file JPPP_A_2365933_SM1365.doc]

**The questionnaire sections :-**

the first section (7 questions) included patients demographic details. The second section of the questionnaire consisted of 5 questions which assessed the prevalence of MTX gastrointestinal adverse effects, where participants gave a (yes) or (no) response.

The third section of the questionnaire comprised of 6 compliance-related statements, and patients were asked to reply using a 5- point Likert scale ranging from (do not agree at all) to (strongly agree). This part assessed several aspects, particularly consistency in MTX utilization, missing doses, folic acid use, reappearance of symptoms after treatment discontinuation and MTX availability in general hospitals pharmacies. The fourth section of the questionnaire consisted of 6 questions which assessed patients' awareness towards MTX, where patients responded using a 5- points Likert scale (strongly agree, agree, undecided, disagree, strongly disagree). In addition, the fifth section of the questionnaire consisted of 9 questions which evaluated MTX functional disability based on the health assessment questionnaire (HAQ)..
